# P-1950. An estimate of pediatric lives saved due to non-pharmacologic interventions during the early COVID-19 pandemic

**DOI:** 10.1093/ofid/ofae631.2109

**Published:** 2025-01-29

**Authors:** Jeremy Samuel Faust, Alexander Chen, Benjamin Renton, Chengan Du, Shu-Xia Li, Zhenqiu Lin, Harlan M Krumholz

**Affiliations:** Brigham and Women’s Hospital, Boston, Massachusetts; Harvard College, Cambridge, Massachusetts; Ontos Analytics, Providence, Rhode Island; Yale School of Medicine, New Haven, Connecticut; Yale School of Medicine, New Haven, Connecticut; Yale School of Medicine, New Haven, Connecticut; Yale University School of Medicine, New Haven, Connecticut

## Abstract

**Background:**

While the number of pediatric deaths attributed directly to COVID-19 is well-characterized, changes in all-cause childhood mortality during the pandemic remain largely unexplored. Quantification of the differences between observed and expected all-cause pediatric deaths during the early pandemic offers insight into the effectiveness of pandemic mitigation strategies for children, a unique group in whom all-cause mortality was not unexpectedly higher due to SARS-CoV-2.

**Table 1.**
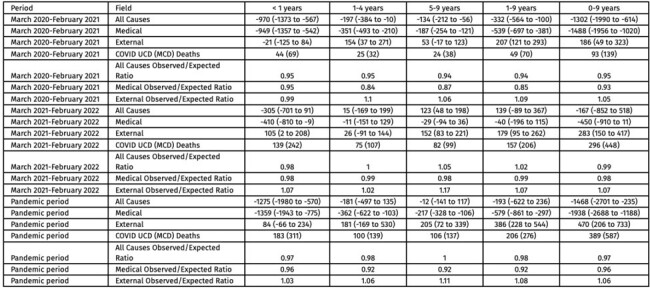
Excess mortality by manner of death, age, and period, March 2020-February 2022.

**Methods:**

To quantify changes in pediatric mortality during the first year of the COVID-19 pandemic, when intense pandemic mitigation measures were maximally implemented.

Using 5 years of pre-pandemic all-cause mortality and population figures (CDC WONDER), we modeled expected monthly mortality in US children for ages ≤9 for all-cause, medical manner, and external manner of death. Overall estimates were assembled from component models of three age groups (< 1, 1-4, 5-9 years).

Expected and observed medical ("natural") deaths, by age grouping, March 2020-February 2022.

**Figure:**
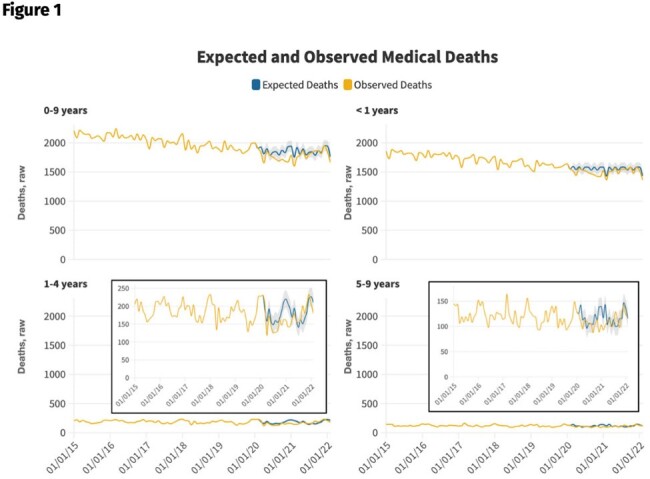

Monthly observed raw deaths are shown in yellow. Modeled expected deaths during the Covid-19 pandemic period are shown in blue. Medical manner of deaths include all ICD-10 classifications that appear in CDC WONDER, except ICD chapters V01-Y89. 95% confidence intervals are indicated by the gray shaded regions.

**Results:**

During the first year of the COVID-19 pandemic (March 2020-February 2021) in the US, a 7% decrease (1,488 fewer deaths; 95% CI -1,956 to -1,020) in medical mortality (“natural causes”) occurred among children ages 0-9, including a 5% decrease among infants < 1 year, and a 15% decrease among children ages 1-9. During the first two full years, there were 1,938 fewer medical deaths than expected (95% CI -2,688-1,188). The usual expected surge in winter medical deaths, particularly among children ages >1 year was absent during the first year. Smaller increases in external (“non-natural causes”) mortality were also observed during the study period, which offset some of the overall decrease in pediatric deaths averted. In total, 1,302 fewer all-cause pediatric deaths (95% CI -1,990 to -614) occurred in the first 12 months of the pandemic and 1,468 fewer all-cause pediatric deaths (95% CI -2,701 to -235) than expected occurred in the United States during the first 24 months of the COVID-19 pandemic.

Expected and observed external ("unnatural") deaths, by age grouping, March 2020-February 2022.

**Figure:**
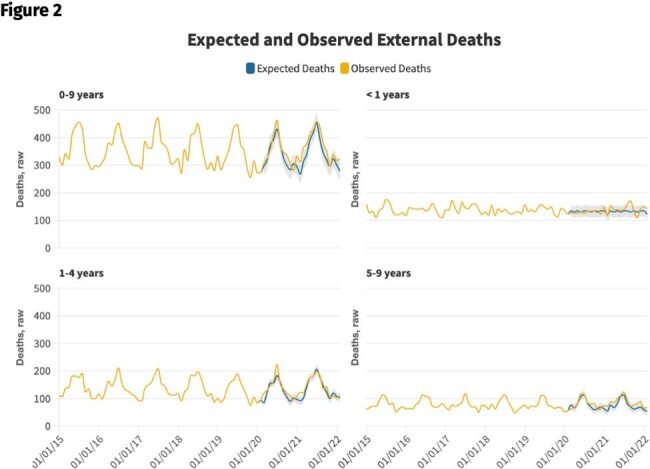

Monthly observed raw deaths are shown in yellow. Modeled expected deaths during the Covid-19 pandemic period are shown in blue. External manner of deaths include ICD-10 classifications within chapters V01-Y89. 95% confidence intervals are indicated by the gray shaded regions.

**Conclusion:**

These findings provide compelling evidence that early and intense pandemic mitigation measures were associated with a measurably favorable overall effect on pediatric all-cause and medical mortality in the US.

Cumulative all-cause excess mortality, external manner of death, and external manner of death, US residents ages 0-9, March 2020-February 2022.

**Figure:**
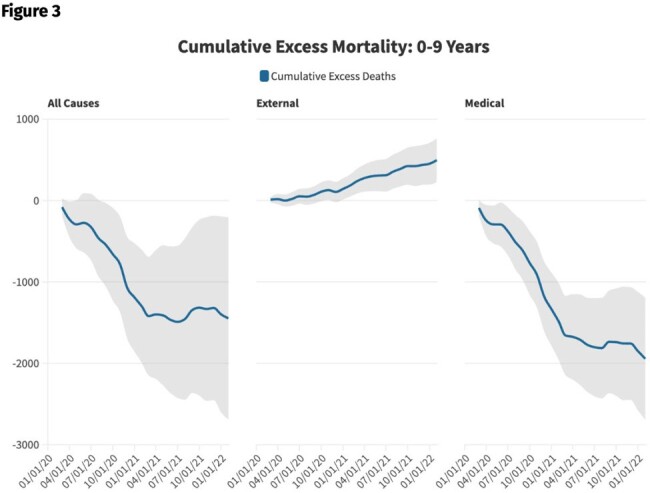

Cumulative raw excess deaths (monthly data) are shown for all-causes (left panel), external manner of death (“unnatural”) causes (middle panel), and medical manner of death (“natural”) causes (right panel). 95% confidence intervals are indicated by the gray shaded regions.

**Disclosures:**

All Authors: No reported disclosures

